# Intrapleural urokinase directly under medical thoracoscopy for the diagnosis of tuberculous pleurisy

**DOI:** 10.1002/rcr2.498

**Published:** 2019-11-07

**Authors:** Satoshi Terashita, Hiroaki Kawachi, Tomoko Tajiri, Susumu Noguchi, Tatsuyoshi Ikeue, Takakazu Sugita

**Affiliations:** ^1^ Department of Respiratory Medicine Japanese Red Cross Wakayama Medical Center Wakayama Japan

**Keywords:** Intrapleural fibrinolysis, medical thoracoscopy, pleural effusions, tuberculous pleurisy, urokinase

## Abstract

Medical thoracoscopy, also called “local anesthetic thoracoscopy” and “pleuroscopy,” is a minimally invasive single‐port endoscopic technique that provides direct visualization of the pleural surfaces and channels to conduct diagnostic and therapeutic procedures. However, this technique is not helpful when substantial fibrous adhesions exist. We reported the first case of intrapleural urokinase directly under medical thoracoscopy for the diagnosis of malignant pleural mesothelioma with severe multiloculated pleural effusions in 2019. This is the second report regarding the efficacy of intrapleural urokinase directly under medical thoracoscopy for the diagnosis of multiloculated pleural effusions. Urokinase‐induced intrapleural fibrinolysis, which removed the fibrous septa, consequently improved the field of view under endoscopy within only 10 min. Fibrinolytic effect appeared very rapidly. This technique is available for tuberculous pleurisy with severe multiloculated pleural effusion.

## Introduction

Loculated pleural effusions occur most commonly in association with conditions that cause intense pleural inflammation, such as empyema, haemothorax, or tuberculosis 1. Furthermore, pleural fluid loculations or trapped lungs frequently occur in patients with malignant pleural effusions. In these patients, augmented procoagulant and depressed fibrinolytic activity contribute to fibrin deposition within the pleural space 2. These conditions obstruct the field of view under medical thoracoscopy and thereby make it difficult to conduct a pleural biopsy.

We reported the first case of intrapleural urokinase directly under medical thoracoscopy for the diagnosis of malignant pleural mesothelioma with severe multiloculated pleural effusions in 2019 3. This is the second report regarding the efficacy of intrapleural urokinase directly under medical thoracoscopy for the diagnosis of multiloculated pleural effusions. This technique is available for tuberculous pleurisy with severe multiloculated pleural effusion.

## Case Report

**Figure 1:**
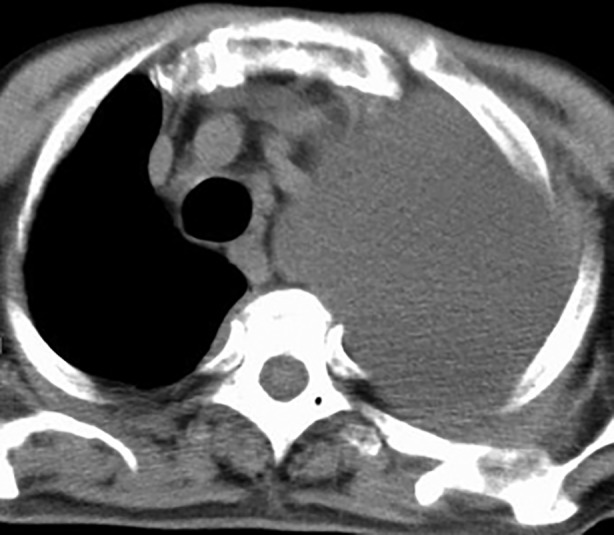
The patient's computed tomography scan of the chest showed large left‐sided pleural effusions.

An 88‐year‐old man with a medical history of diabetes mellitus presented to the outpatient department with a 2‐week history of chest pain and cough. His vital signs were normal, and SpO_2_ was 94% on room air. His physical examination was remarkable for heavily decreased breath sounds in the left lung. The patient's chest radiograph and a computed tomography scan of the chest showed large left‐sided pleural effusions (Fig. 1). The adenosine deaminase (ADA) level in pleural fluid was 67.5 IU/L. Interferon‐Gamma release assay was negative. Diagnostic thoracocentesis was performed twice, but bacterial cultures showed no growth, and acid‐fast bacilli smears were negative. The cause of the pleural effusions was unknown.

**Figure 2:**
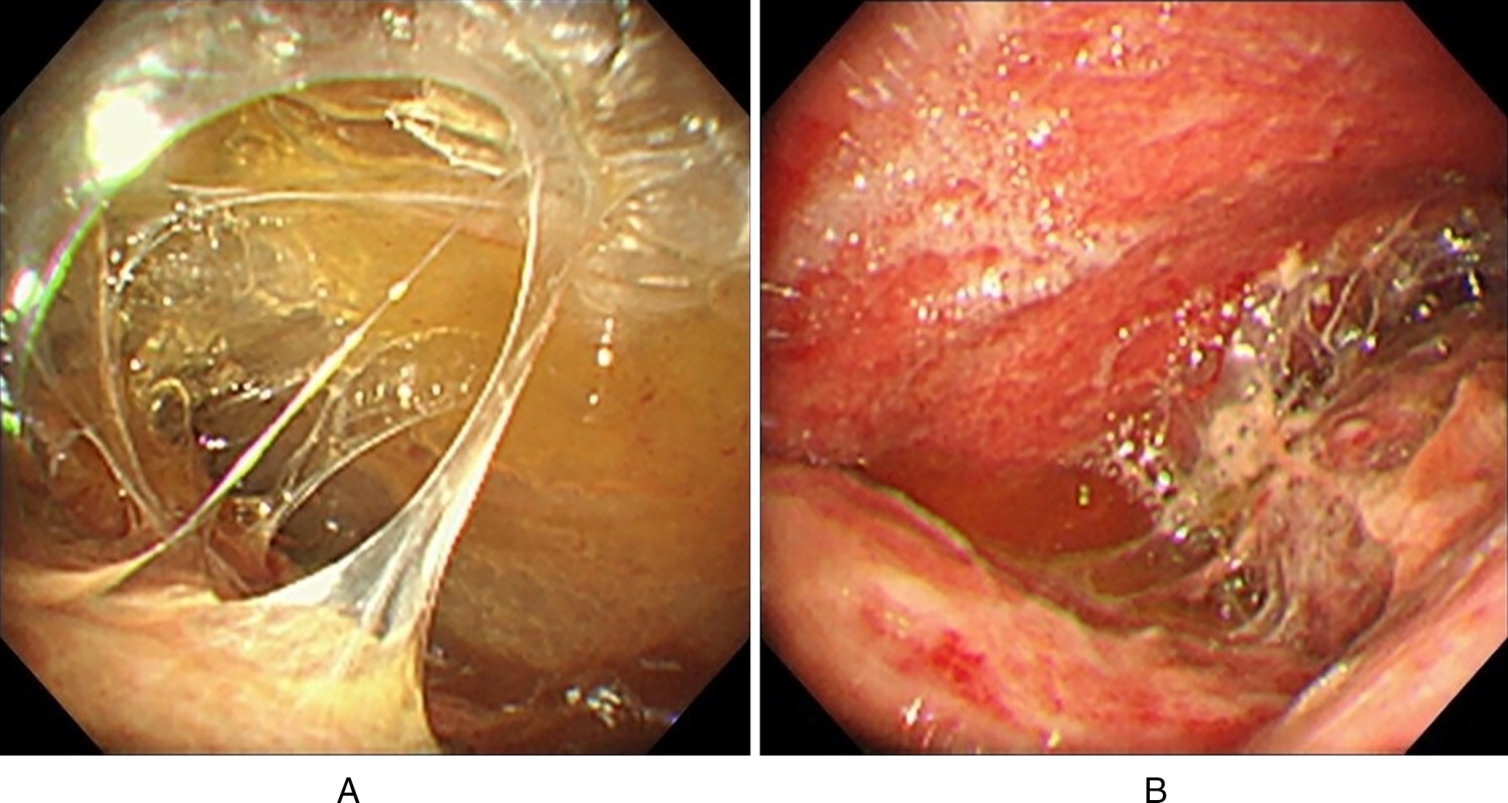
Medical thoracoscopic findings. (A) Owing to the massive intrapleural fibrin nets, we were unable to sufficiently observe the pleural space. (B) Approximately 10 min after administering urokinase, intrapleural urokinase fibrinolysis was observed, and the intrapleural fibrous septa had vanished. As a result, we were able to identify the parietal pleura, which showed multiple white small nodules.

Because of massive pleural effusions, the insertion of a chest drain was performed first, and subsequently, medical thoracoscopy was conducted on the following day. A thoracoscope demonstrated remarkable intrapleural fibrin deposition. Owing to the massive fibrin nets, we were unable to sufficiently observe the pleural space (Fig. 2A). Therefore, we attempted to inject urokinase into the multiloculated pleural space.

A dose of 60,000 IU urokinase reconstituted in 100 mL of 0.9% saline was administered through the biopsy port of the semirigid thoracoscope. Approximately 10 min after administering urokinase, intrapleural urokinase fibrinolysis was observed, and the intrapleural fibrous septa had vanished in the vicinity of the thoracoscope. As a result, the field of view under endoscopy became clear, and we were able to identify the parietal pleura, which showed multiple white small nodules (Fig. 2B). A biopsy of these nodules was conducted. After the biopsy, we completed the medical thoracoscopy without any complications. No bleeding, fever, anaphylaxis, or allergic reactions were noted. The examination time was 62 min. In the biopsy specimens, the acid‐fast bacilli smears were positive, and *Mycobacterium tuberculosis* polymerase chain reaction (PCR) assay was positive. Furthermore, cultures showed the growth of *M. tuberculosis*. We consequently diagnosed tuberculous pleurisy.

## Discussion

Medical thoracoscopy, also called “local anesthetic thoracoscopy” and “pleuroscopy,” is a minimally invasive single‐port endoscopic technique that provides direct visualization of the pleural surface and allows for both diagnostic and therapeutic procedures 4. Its utility, however, is limited when fibrous adhesions are substantial 4. In fact, based on British Thoracic Society guidelines, lungs adherent to the chest wall throughout the hemithorax are an absolute contraindication to this procedure 5. Many reports, however, have focused on the efficacy of using fibrinolytic agents to break down pleural loculations, primarily in the setting of pleural infection. Furthermore, several small series have also reported the use of intrapleural fibrinolysis for the management of loculated malignant effusions 2.

However, the application of intrapleural urokinase directly through a medical thoracoscope for loculated pleural effusions had not been documented until our first report. We reported the first case of the successful diagnosis of malignant pleural mesothelioma with severe multiloculated pleural effusion by using intrapleural urokinase directly under medical thoracoscopy in 2019 3. This is the second report regarding the efficacy of intrapleural urokinase directly under medical thoracoscopy for the diagnosis of multiloculated pleural effusions. This technique was available for tuberculous pleurisy with severe multiloculated pleural effusion. In our patient, intrapleural urokinase administration through the biopsy port of a semi‐rigid thoracoscope at a dose of 60,000 IU (diluted in 100 mL of normal saline) induced fibrinolysis and dissolution of multiloculated pleural effusions within only 10 min. Although the appropriate dose of urokinase is still incompletely understood, the dose and method of intrapleural urokinase directly under medical thoracoscopy were the same as our first report. The field of view under medical thoracoscopy subsequently became clear to an extent that allowed the successful diagnosis based on parietal pleural biopsy findings.

Intrapleural urokinase directly under medical thoracoscopy for the diagnosis of multiloculated pleural effusions seem to be convenient. Moreover, the fibrinolytic effect occurs very rapidly. This procedure may expand the diagnostic capability of medical thoracoscopy.

### Disclosure Statement

Appropriate written informed consent was obtained for publication of this case report and accompanying images.
